# Obesity increases the risk of erosive esophagitis but metabolic unhealthiness alone does not: a large-scale cross-sectional study

**DOI:** 10.1186/s12876-018-0814-y

**Published:** 2018-06-08

**Authors:** Myong Ki Baeg, Sun-Hye Ko, Seung Yeon Ko, Hee Sun Jung, Myung-Gyu Choi

**Affiliations:** 1 Department of Internal Medicine, International St. Mary’s Hospital, College of Medicine, Catholic Kwandong University, Incheon, 22711 South Korea; 2 Department of Internal Medicine, Inje University Haeundae Paik Hospital, Inje University College of Medicine, 875 Haeundaero, Haeundae-Gu, Busan, 612-896 South Korea; 3 Department of Surgery, Sacred Heart Hospital, Hallym University, Gyeonggi-do, Anyang-si, 14068 South Korea; 4 Department of Health Promotion, Seoul St. Mary’s Hospital, College of Medicine, The Catholic University of Korea, Seoul, 06591 South Korea; 5 Department of Internal Medicine, Seoul St. Mary’s Hospital, College of Medicine, The Catholic University of Korea, Seoul, 06591 South Korea

**Keywords:** Erosive esophagitis, Gastroesophageal reflux disease, Metabolic health status, Obesity, Wildman criteria

## Abstract

**Background:**

Obesity is a known risk factor for erosive esophagitis (EE) and metabolic unhealthiness has been implicated in EE pathogenesis. However, obesity and metabolic unhealthiness are not synonymous and the associations between obesity, metabolic health, and EE are unclear. Therefore, our aim was to investigate the relationship between EE, obesity, and metabolic health.

**Methods:**

We performed a retrospective cross-sectional study of subjects undergoing health screening at a university hospital. Subjects were classified into 4 groups based on metabolic and obesity criteria: metabolically healthy nonobese (MHNO), metabolically healthy obese (MHO), metabolically unhealthy nonobese (MUNO), and metabolically unhealthy obese (MUO). Multivariable analysis was used to identify EE risk factors with MHNO subjects as reference. To determine if there were synergistic interactions between metabolic health and obesity status, the Rothman’s synergy index and attributable proportion of risk were also calculated.

**Results:**

We included 10,338 subjects (5448 MHNO, 1605 MHO, 1600 MUNO, 1685 MUO). The prevalence of EE was 6.5% in MHNO, 12.6% in MHO, 9.3% in MUNO, and 14.3% in MUO. EE risk was increased significantly by obesity (MHO: OR, 1.589, 95% CI, 1.314–1.921, *P* < 0.001; MUO: OR, 1.734, 95% CI, 1.441–2.085, *P* < 0.001), but not in MUNO subjects (OR, 1.224, 95% CI, 0.991–1.511, *P* = 0.060). Male sex, blood leukocyte count, alcohol, and smoking significantly increased EE risk, but *H. pylori* infection was protective. Replacement of obesity with abdominal obesity gave similar results. The Rothman’s synergy index was 0.920 (95% CI, 0.143–5.899) and the attributable proportion of risk was − 0.051 (95% CI, − 1.206–1.105), indicating no interaction between metabolic and obesity status on EE risk.

**Conclusions:**

We demonstrated that obesity increased the risk of EE, regardless of metabolic health status. However, EE risk was not significantly increased in MUNO subjects, suggesting that metabolic unhealthiness may not be involved in EE pathogenesis. As observational cross-sectional studies cannot prove causality, prospective longitudinal studies involving obesity and metabolic treatment should be performed to further investigate the association between obesity, metabolic health, and EE risk.

## Background

Gastroesophageal reflux disease (GERD) is a multifactorial disease that has genetic, physiologic and environmental risk factors [1]. One risk factor that has attracted great interest is obesity [2], of which the epidemic increase has paralleled the global increase in GERD [3]. The potential mechanisms linking obesity with GERD are pathophysiologic changes brought on by increased intra-abdominal pressure and metabolic unhealthiness associated with proinflammatory cytokine production and the insulin/insulin growth factor pathway [1, 4]. Although obesity has often been regarded as being synonymous with metabolic unhealthiness, not all obese people are metabolically unhealthy and one-third of metabolically unhealthy people are of normal weight [5, 6]. However, the association between obesity, metabolic health, and GERD has not been investigated adequately.

Endoscopically visible breaks in the gastroesophageal junction are a reliable sign of GERD. This is clinically important because healing of such endoscopically confirmed erosive esophagitis (EE) can be regarded as a surrogate for successful therapy and correlates well with symptomatic relief [7]. EE is associated with potentially serious complications such as Barrett’s esophagus (BE) and esophageal adenocarcinoma (EAC) [8, 9], which have also been linked with obesity and metabolic unhealthiness [10–12]. Therefore, the aim of our study was to investigate the relationship between EE, obesity, and metabolic health status.

## Methods

### Study population

We performed a retrospective cross-sectional study of subjects who underwent routine health screening from March 2009 to July 2014 at the Center for Health Promotion of Seoul St. Mary’s Hospital (Seochogu, Seoul, South Korea). Subjects underwent health screening voluntarily or as part of annual/biannual employee check-ups. Such check-ups cover about 40–50% of the Korean population. Those who underwent screening esophagogastroduodenoscopy and for whom fasting serum insulin results were available were included in this study. Those who underwent multiple visits had only the first set of endoscopy included. We excluded subjects who 1) had a history of current or previous malignancies, 2) had a history of upper gastrointestinal surgery, and 3) were missing medical or social records or anthropometric/laboratory findings. This study was approved by the Institutional Review Board of Seoul St. Mary’s Hospital, which permitted the study without informed consent requirements because it was a retrospective study using blinded subject identities (KC14RISI0574).

### Data collection

Physical characteristics including weight, height, waist circumference, and blood pressure were measured by trained medical personnel. Blood pressure was measured using an appropriately sized cuff with the subject in a sitting position after at least 10 min of rest. Waist circumference was measured at the midline between the lowest rib and the iliac crest.

Blood samples were taken after an overnight fast of at least 12 h. White blood cell (WBC) counts were analyzed using a Sysmex-XE2100 automated blood cell analyzer (Sysmex, Kobe, Japan). Fasting plasma glucose (FPG), total cholesterol, triglyceride, high-density lipoprotein (HDL) cholesterol, and low-density lipoprotein cholesterol levels were measured using a Hitachi 7600 automated analyzer (Hitachi Co., Tokyo, Japan). Glycated hemoglobin (HbA1c) was measured using a Tosoh HLC-723 HbG7 analyzer (Tosoh Bioscience Ltd., Redditch, UK). *Helicobacter pylori-*specific immunoglobulin G concentration was measured using the Immulite 2000 XPi platform (Siemens Healthcare Diagnostics, Erlangen, Germany).

### Esophagogastroduodenoscopy

Esophagogastroduodenoscopy (Olympus GIF-H260; Olympus Ltd., Tokyo, Japan) was performed in subjects who had fasted overnight by endoscopists who were board-accredited gastroenterologists and certified as experts by the Korean Society of Gastrointestinal Endoscopy. EE was defined according to the Los Angeles Classification [13]. All endoscopy results were reassessed visually by two authors who were blinded to the initial endoscopy records.

### Definitions

Body mass index was calculated as weight divided by height squared (kg/m^2^). Obesity was defined according to the World Health Organization Criteria for East Asians (> 25 kg/m^2^) [14]. Abdominal obesity was defined as waist circumference ≥ 90 cm in men and ≥ 80 cm in women, which are the modified criteria for the Asian population [15]. Insulin resistance was computed by the homeostasis model assessment of insulin resistance (HOMA-IR) as follows: fasting insulin (pmol/L) × fasting glucose (mmol/L)/22.5 [16]. Metabolic health status was determined by the modified Wildman criteria, which were as follows: (1) systolic blood pressure ≥ 130 mmHg or diastolic blood pressure ≥ 85 mmHg or use of antihypertensive medication, (2) triglyceride levels ≥ 1.7 mmol/L or use of lipid-lowering drugs, (3) FPG ≥ 5.5 mmol/L or use of antidiabetes therapy, (4) HDL cholesterol levels < 1.0 mmol/L in men and < 1.3 mmol/L in women, and (5) HOMA-IR > 90th percentile in our population (≥ 3.17) [6, 17, 18]. Subjects were defined as metabolically healthy if they met ≤ 1 of the modified Wildman criteria and metabolically unhealthy if they met ≥ 2 of the criteria. Based on the modified Wildman and obesity criteria, the subjects were classified as (1) metabolically healthy nonobese (MHNO), (2) metabolically healthy obese (MHO), (3) metabolically unhealthy nonobese (MUNO), and (4) metabolically unhealthy obese (MUO).

### Statistical analysis

Clinical characteristics and parameters were expressed as mean ± standard deviation or numbers (percentage). Categorical variables were analyzed by Pearson’s chi-square test, and continuous variables by analysis of variance. *P* values < 0.05 were considered significant. Multivariable regression analysis was performed to identify risk factors for EE. Odds ratios (ORs) and 95% confidence intervals (CIs) for EE were calculated for the MHO, MUNO, and MUO groups using the MHNO group as the reference category. A separate analysis was performed, replacing obesity with abdominal obesity to investigate its effects. In addition, we analyzed the interaction between metabolic health and obesity status by calculating the Rothman’s synergy index and the attributable proportion of risk [19, 20]. If metabolic health and obesity had an interaction to increase the risk of EE, the synergy index would be greater than 1.0, whereas if there were no additive effects, the value would be below 1. The attributable proportion of risk gives an estimate of the proportion of EE cases that are attributable to any interaction between metabolic health and obesity status beyond each factor alone. If there were any positive interaction, the attributable proportion of risk would be greater than 0 [19].

## Results

**Fig. 1:**
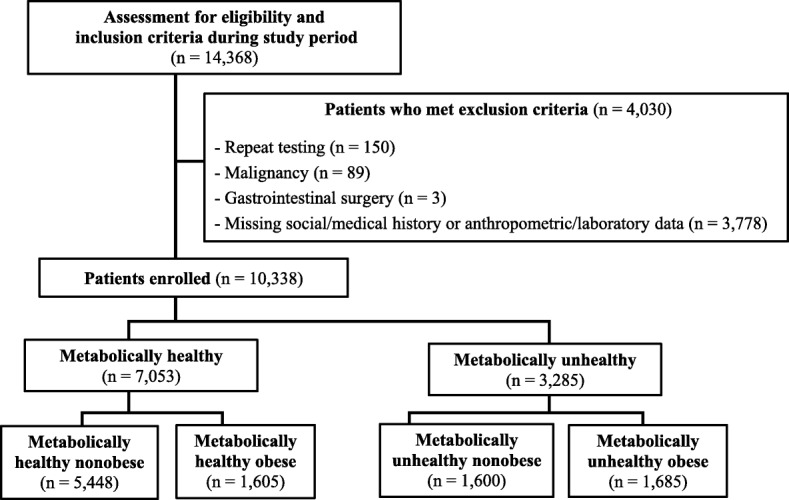
Flow chart of the study design

During routine health screening in the study period, 14,368 Koreans underwent screening esophagogastroduodenoscopy and fasting insulin measurement. Of these, 4030 were excluded for the following reasons: (1) 150 because of repeat testing, (2) 89 with malignancies, (3) three with prior upper gastrointestinal surgery, and (4) 3788 with missing social or medical records or anthropometric/laboratory data. Of the 10,338 subjects included in the study, there were 5448 in the MHNO group, 1605 in the MHO group, 1600 in the MUNO group, and 1685 in the MUO group (Fig. 1).

The prevalence of EE was 6.5% in the MHNO group, 12.6% in the MHO group, 9.3% in the MUNO group, and 14.3% in the MUO group. There was a significantly higher proportion of males in the obese groups. Indices related to glucose metabolism such as FPG, HbA1c, insulin, and HOMA-IR were lowest in the MHNO group and successively increased in the MHO, MUNO, and MUO groups. The percentage of subjects who drank coffee or alcohol was significantly higher in the obese groups. Other characteristics of the four groups are listed in Table 1.

**Table 1:** Characteristics of subjects by metabolic obesity status

	MHNO (*n* = 5448)	MHO (*n* = 1605)	MUNO (*n* = 1600)	MUO (*n* = 1685)	*P*
Age (years)	49.5 ± 11.4	51.6 ± 11.1	56.5 ± 10.3	53.9 ± 11.2	< 0.001
Male (n, %)	2339 (42.9%)	1074 (66.9%)	921 (57.6%)	1204 (71.5%)	< 0.001
Diabetes (n, %)	153 (2.8%)	50 (3.1%)	418 (26.1%)	467 (27.7%)	< 0.001
Hypertension (n, %)	582 (10.7%)	328 (20.4%)	616 (38.5%)	682 (40.5%)	< 0.001
BMI (kg/m^2^)	21.7 ± 2.0	26.8 ± 1.7	22.9 ± 1.6	27.6 ± 2.3	< 0.001
Abdominal obesity (n, %)	1454 (26.7%)	1127 (70.2%)	574 (35.9%)	1320 (78.3%)	< 0.001
Coffee (n, %)	3664 (67.3%)	1156 (72.0%)	1025 (64.1%)	1240 (73.6%)	< 0.001
Alcohol (n, %)	2337 (42.9%)	932 (58.1%)	735 (45.9%)	949 (56.3%)	< 0.001
Smoking (n, %)	1883 (34.6%)	705 (43.9%)	715 (44.7%)	886 (52.6%)	< 0.001
*H.pylori* seropositive (n, %)	2757 (50.6%)	839 (52.3%)	863 (53.9%)	922 (54.7%)	0.008
WBC count (× 10^9^/L)	5.4 ± 1.7	5.7 ± 1.6	6.0 ± 1.8	6.3 ± 1.7	< 0.001
FPG (mmol/L)	4.9 ± 0.8	5.1 ± 0.7	6.1 ± 1.8	6.2 ± 1.6	< 0.001
HbA1c (%)	5.4 ± 0.5	5.5 ± 0.5	6.0 ± 1.1	6.1 ± 1.0	< 0.001
Insulin (pmol/L)	34.7 ± 21.5	50.0 ± 27.1	54.9 ± 38.9	80.6 ± 61.8	< 0.001
HOMA-IR	1.1 ± 0.7	1.6 ± 0.9	2.2 ± 2.0	3.2 ± 2.5	< 0.001
Total cholesterol (mmol/L)	5.1 ± 0.9	5.2 ± 0.9	5.2 ± 1.0	5.2 ± 1.1	< 0.001
Triglyceride (mmol/L)	0.9 ± 0.5	1.1 ± 0.6	1.8 ± 1.1	2.0 ± 1.3	< 0.001
HDL-cholesterol (mmol/L)	1.5 ± 0.3	1.3 ± 0.3	1.2 ± 0.3	1.1 ± 0.3	< 0.001
LDL-cholesterol (mmol/L)	3.1 ± 0.8	3.3 ± 0.8	3.1 ± 0.9	3.2 ± 0.9	< 0.001
EE (n, %)	356 (6.5%)	202 (12.6%)	149 (9.3%)	241 (14.3%)	< 0.001
EE grade (n, %)					< 0.001
A	310 (5.7%)	172 (10.7%)	127 (7.9%)	186 (11.0%)	
B	37 (0.1%)	27 (1.7%)	19 (1.2%)	45 (2.7%)	
C or D	9 (0.2%)	3 (0.2%)	3 (0.2%)	10 (0.6%)	

Data are presented as either mean ± standard deviation or as number (%)

*BMI* body mass index, *EE* erosive esophagitis, *FPG* fasting plasma glucose, *HbA1c* glycated hemoglobin, *HDL* high density lipoprotein, *HOMA-IR* homeostasis model assessment of insulin resistance, *MHNO* metabolic healthy nonobese, *MHO* metabolic healthy obese, *MUNO* metabolic unhealthy nonobese, *MUO* metabolic unhealthy obese, *LDL* low density lipoprotein, *TC* total cholesterol, *WBC* white blood cell

Characteristics of the subjects based on EE status are shown in Table 2. Subjects with EE were significantly more likely to be male and obese, and included significantly more subjects who drank coffee or alcohol and had a smoking history. Those with EE were significantly less likely to be seropositive for *H. pylori*.

**Table 2:** Characteristics of subjects with or without erosive esophagitis

	EE (−) (*n* = 9390)	EE (+) (*n* = 948)	*P*
Age (years)	51.7 ± 11.4	51.2 ± 11.6	0.193
Male (n, %)	4780 (50.9%)	758 (80.0%)	< 0.001
Diabetes (n, %)	963 (10.3%)	125 (13.2%)	0.005
Hypertension (n, %)	1960 (20.9%)	248 (26.2%)	< 0.001
Obesity (n, %)	2847 (30.3%)	443 (46.7%)	< 0.001
Abdominal obesity (n, %)	4040 (43.0%)	432 (45.6%)	0.119
Metabolic unhealthiness (n, %)	2895 (30.8%)	390 (41.1%)	< 0.001
Metabolic obesity status (n, %)			< 0.001
Metabolic healthy nonobese	5092 (54.2%)	356 (37.6%)	
Metabolic healthy obese	1403 (14.9%)	202 (21.3%)	
Metabolic unhealthy nonobese	1451 (15.5%)	149 (15.7%)	
Metabolic unhealthy obese	1444 (15.4%)	241 (25.4%)	
Metabolic abdominal obesity status (n, %)			< 0.001
Metabolic healthy abdominal nonobese	4122 (43.9%)	351 (37.0%)	
Metabolic healthy abdominal obese	2373 (25.3%)	207 (21.8%)	
Metabolic unhealthy abdominal nonobese	1228 (13.1%)	165 (17.4%)	
Metabolic unhealthy abdominal obese	1667 (17.8%)	225 (23.7%)	
Smoking (n, %)	3634 (38.7%)	555 (58.5%)	< 0.001
Alcohol (n, %)	4321 (46.0%)	632 (66.7%)	< 0.001
Coffee (n, %)	6386 (68.0%)	699 (73.7%)	< 0.001
Laboratory findings			
*Helicobacter pylori* seropositive (n, %)	5080 (54.1%)	301 (31.8%)	< 0.001
White blood cell count (× 10^9^/L)	5.7 ± 1.7	6.1 ± 1.8	< 0.001
Fasting plasma glucose (mmol/L)	5.3 ± 1.3	5.5 ± 1.6	< 0.001
Glycated hemoglobin (%)	5.6 ± 0.7	5.8 ± 0.9	< 0.001
Insulin (pmol/L)	6.7 ± 5.5	7.8 ± 5.7	< 0.001
HOMA-IR	1.65 ± 1.57	2.01 ± 2.05	< 0.001
Total cholesterol (mmol/L)	5.2 ± 1.0	5.2 ± 0.9	0.719
Triglyceride (mmol/L)	1.2 ± 0.9	1.5 ± 1.1	< 0.001
High density lipoprotein cholesterol (mmol/L)	1.4 ± 0.3	1.3 ± 0.3	< 0.001
Low density lipoprotein cholesterol (mmol/L)	3.2 ± 0.8	3.2 ± 0.8	0.628

Data are presented as either mean ± standard deviation or as number (%)

*EE* erosive esophagitis, *HOMA-IR* homeostasis model assessment of insulin resistance

Univariable analysis of EE risk factors found that male sex, higher WBC count, higher glucose metabolism indices, drinking coffee or alcohol, or smoking significantly increasedEE risk. Protective factors for EE were higher HDL cholesterol levels and *H. pylori* seropositivity (Table 3).

**Table 3:** Univariable risk factors of erosive esophagitis

	Odds ratio	95% Confidence interval	*P*
Age	0.996	0.990–1.002	0.193
Male	3.848	3.265–4.534	< 0.001
Diabetes	1.329	1.089–1.623	0.005
Hypertension	1.343	1.152–1.566	< 0.001
Obesity	2.024	1.768–2.316	< 0.001
Coffee	1.321	1.135–1.536	< 0.001
Alcohol	2.346	2.038–2.701	< 0.001
Smoking	2.237	1.953–2.562	< 0.001
*Helicobacter pylori* seropositivity	0.395	0.342–0.455	< 0.001
White blood cell count	1.145	1.105–1.185	< 0.001
Fasting plasma glucose	1.006	1.004–1.008	< 0.001
Glycated hemoglobin	1.206	1.121–1.298	< 0.001
Insulin	1.027	1.017–1.037	< 0.001
HOMA-IR	1.1	1.065–1.137	< 0.001
Total cholesterol	1	0.999–1.002	0.719
Triglyceride	1.003	1.003–1.004	< 0.001
High density lipoprotein cholesterol	0.979	0.974–0.985	< 0.001
Low density lipoprotein cholesterol	1.001	0.998–1.003	0.628
Metabolic Obesity status			
Metabolic healthy nonobese	1		
Metabolic healthy obese	2.059	1.716–2.472	< 0.001
Metabolic unhealthy nonobese	1.469	1.203–1.794	< 0.001
Metabolic unhealthy obese	2.387	2.007–2.840	< 0.001
Metabolic abdominal obesity status			
Metabolic healthy abdominal nonobese	1		
Metabolic healthy abdominal obese	1.024	0.856–1.225	0.792
Metabolic unhealthy abdominal nonobese	1.578	1.298–1.919	< 0.001
Metabolic unhealthy abdominal obese	1.585	1.328–1.892	0.001

*HOMA-IR* homeostasis model assessment of insulin resistance

Multivariable analysis found that compared with the reference MHNO group, EE risk was significantly higher in the obese groups (MHO: OR, 1.589, 95% CI, 1.314–1.921, *P* < 0.001; MUO: OR, 1.734, 95% CI, 1.441–2.085, *P* < 0.001), but not in the MUNO group (OR, 1.224, 95% CI, 0.991–1.511, *P* = 0.060). Other predictive factors for EE were male sex, higher WBC counts, alcohol, and smoking history while *H. pylori* seropositivity remained protective (Table 4). When obesity was replaced in the analysis with abdominal obesity, the risk factors for EE remained similar. EE risk was significantly higher in the groups with abdominal obesity but not in the metabolically healthy abdominally nonobese group (Table 4). Analyzing obesity and metabolic health separately by multivariable analysis gave similar results, with obesity being a significant risk factor for EE (OR, 1.521, 95% CI, 1.314–1.761, *P* < 0.001), but metabolic health was not significant (OR, 1.154, 95% CI, 0.992–1.342, *P* = 0.064).

**Table 4:** Multivariable risk factors of erosive esophagitis according to metabolic obesity status or metabolic abdominal obesity status

	Odds ratio	95% CI	*P*	Odds ratio	95% CI	*P*
Age	1.002	0.996–1.008	0.540	1	0.994–1.006	0.982
Male	2.691	2.196–3.297	< 0.001	3.358	2.727–4.136	< 0.001
Coffee	1.075	0.918–1.258	0.369	1.087	0.929–1.273	0.298
Alcohol	1.338	1.138–1.574	< 0.001	1.346	1.145–1.583	< 0.001
Smoking	1.181	1.007–1.385	0.041	1.171	0.999–1.373	0.052
*Helicobacter pylori* seropositivity	0.362	0.313–0.419	< 0.001	0.364	0.315–0.422	< 0.001
White blood cell count	1.071	1.029–1.114	0.001	1.068	1.027–1.111	0.001
Metabolic Obesity Status						
Metabolic healthy nonobese	1					
Metabolic healthy obese	1.589	1.314–1.921	< 0.001			
Metabolic unhealthy nonobese	1.224	0.991–1.511	0.06			
Metabolic unhealthy obese	1.734	1.441–2.085	< 0.001			
Metabolic Abdominal Obesity Status						
Metabolic healthy abdominal nonobese				1		
Metabolic healthy abdominal obese				1.585	1.308–1.920	< 0.001
Metabolic unhealthy abdominal nonobese				1.186	0.966–1.455	0.103
Metabolic unhealthy abdominal obese				1.807	1.496–2.182	< 0.001

*CI* confidence interval

We calculated the Rothman’s synergy index and attributable proportion of risk to identify any interaction between metabolic health and obesity on EE. The Rothman’s synergy index and the attributable proportion of risk was 0.920 (< 1, 95% CI, 0.143–5.899) and − 0.051 (< 0, 95% CI, − 1.206–1.105), respectively, which suggest that there were no synergistic effects or increased risks from the interaction between them.

## Discussion

This study demonstrated that obesity increases the risk of EE, regardless of metabolic health status. Nonobese metabolically unhealthy subjects did not have a significant increase in EE risk while there was a significant increase in those who were obese. These findings were consistent when abdominal obesity replaced obesity in the analysis. Our study shows that obesity or abdominal obesity plays a significant role in EE risk, whereas metabolic health may not.

Our study reinforced the classic view that obesity or abdominal obesity increases EE risk [1, 21]. This is most likely because of the increased intraabdominal pressure, increased transient lower esophageal sphincter relaxation and anatomic disruption of the esophagogastric junction brought on by obesity or abdominal obesity [1, 21].

Recently, a new perspective has suggested that GERD is also mediated by a metabolic pathway [4]. This is supported by several studies that have reported a positive association between insulin resistance and GERD [22–24]. However, these studies were limited by their small number of subjects, that they included only obese subjects without a nonobese control group or had potential multicollinearity issues [22–24]. Most importantly, obesity and metabolic unhealthiness were not analyzed independently, which may have confounded the outcome. In our study, EE risk was dependent on obesity but not on metabolic status. This suggests that metabolic unhealthiness by itself may not be a sufficient risk factor for EE, but is subordinate to obesity.

Interestingly, our study also found that increased WBC counts may increase EE risk. The WBC count is a biomarker of systemic inflammation and has been associated with increased overall, cancer-related, and vascular disease-related mortality [25]. Though metabolic unhealthiness was not associated with the risk of EE, the significant increase in WBC count in subjects with EE subjects suggests that inflammatory processes may contribute to EE. This is supported by a recent study that reported that subjects with erosive esophagitis had significantly higher standardized ^18^F-fluorodeoxyglucose uptake values at the esophagogastric junction [26]. It is also supported by studies that have reported that levels of proinflammatory cytokines such as interleukins 1, 6, and 8 were increased in subjects with GERD [27]. However, as both EE and non-EE subjects had WBC counts within the normal range and MUNO subjects had higher WBC counts than MHO subjects, the association between higher WBC counts and increased EE risk cannot be asserted confidently. Studies involving EE and the inflammatory pathway should be performed to further investigate this association.

Our study is limited by its retrospective cross-sectional design, which allows only inferences to be made regarding obesity/metabolic health status and risk of EE. Another limitation is the lack of data regarding inflammatory factors such as interleukin 1, 6, 8, and tumor necrosis factor alpha, which may have shed more light on the association between inflammation and EE. A third limitation is that we only included subjects for whom both insulin and endoscopy results were available, which may have resulted in a selection bias. However, because our study included over 10,000 subjects, we believe that the risk of selection bias is low. Finally, we could not investigate the association between BE or EAC and obesity/metabolic health because the prevalence of BE and EAC is very low in Koreans [28, 29].

The main strength of our study is that this is the first study investigating the risk of EE according to a standardized obesity/metabolic health profile to divide the subjects into four distinct obesity/metabolic groups. This enabled us to distinguish the effects of obesity and metabolic health, which contrasts with previous studies whose findings may have been related to underlying obesity rather than to metabolic unhealthiness [22, 24]. Another strength is the large number of subjects included in our study, with at least 1600 subjects in each subgroup. Though this study was of a retrospective cross-sectional design, we believe that the inclusion of a sufficiently large number of subjects lends credibility to our study results. Lastly, we calculated the Rothman’s synergy index and attributable proportion of risk to determine if there was any interaction between metabolic health and obesity status. This analysis verified our findings in that there were no significant interactions between metabolic health and obesity status, which also suggests that the risk of EE conferred by obesity was independent of metabolic health status.

## Conclusion

Our study found that obesity/abdominal obesity was a risk factor for EE, regardless of metabolic health status. Though EE risk was significantly increased in the MUO group compared with the reference MHNO group, it was not in the MUNO group, which suggests that metabolic health plays a marginal role in EE pathogenesis. Prospective longitudinal studies including body composition and metabolic health analysis as in addition to those involving obesity and metabolic treatment should be performed to further investigate the association between obesity, metabolic health, and EE risk.

## Abbreviations

BE: Barrett’s esophagus
CI: Confidence interval
EAC: Esophageal adenocarcinoma
EE: Erosive esophagitis
FPG: Fasting plasma glucose
GERD: Gastroesophageal reflux disease
HbA1c: Glycated hemoglobin
HOMA-IR: Homeostasis model assessment of insulin resistance
MHNO: Metabolically healthy nonobese
MHO: Metabolically healthy obese
MUNO: Metabolically unhealthy nonobese
MUO: Metabolically unhealthy obese
OR: Odds ratio
WBC: White blood cell
